# Multivariate genomic analysis and optimal contributions selection predicts high genetic gains in cooking time, iron, zinc, and grain yield in common beans in East Africa

**DOI:** 10.1002/tpg2.20156

**Published:** 2021-10-26

**Authors:** Renu Saradadevi, Clare Mukankusi, Li Li, Winnyfred Amongi, Julius Peter Mbiu, Bodo Raatz, Daniel Ariza, Steve Beebe, Rajeev K. Varshney, Eric Huttner, Brian Kinghorn, Robert Banks, Jean Claude Rubyogo, Kadambot H. M. Siddique, Wallace A. Cowling

**Affiliations:** ^1^ The UWA Institute of Agriculture The Univ. of Western Australia Perth Western Australia 6009 Australia; ^2^ UWA School of Agriculture and Environment The Univ. of Western Australia Perth Western Australia 6009 Australia; ^3^ Alliance of Bioversity International & International Center for Tropical Agriculture (CIAT) PO Box 6247 Kampala Uganda; ^4^ Animal Genetics and Breeding Unit Univ. of New England Armidale New South Wales 2351 Australia; ^5^ Tanzania Agricultural Research Institute (TARI) Maruku PO Box 127 Bukoba Kagera Tanzania; ^6^ Alliance of Bioversity International & International Center for Tropical Agriculture (CIAT) Cali Colombia; ^7^ Australian Centre for International Agricultural Research Canberra Australian Capital Territory 2617 Australia; ^8^ Centre of Excellence in Genomics and Systems Biology International Crops Research Institute for the Semi‐Arid Tropics (ICRISAT) Hyderabad 502324 India; ^9^ State Agricultural Biotechnology Centre, Centre for Crop and Food Innovation Murdoch Univ. Murdoch Western Australia 6150 Australia; ^10^ School of Environmental and Rural Science Univ. of New England Armidale New South Wales 2351 Australia; ^11^ Alliance of Bioversity International & International Center for Tropical Agriculture (CIAT) Nairobi Kenya; ^12^ Current address: Vilmorin SA la Menitré France

## Abstract

Common bean (*Phaseolus vulgaris* L.) is important in African diets for protein, iron (Fe), and zinc (Zn), but traditional cultivars have long cooking time (CKT), which increases the time, energy, and health costs of cooking. Genomic selection was used to predict genomic estimated breeding values (GEBV) for grain yield (GY), CKT, Fe, and Zn in an African bean panel of 358 genotypes in a two‐stage analysis. In Stage 1, best linear unbiased estimates (BLUE) for each trait were obtained from 898 genotypes across 33 field trials in East Africa. In Stage 2, BLUE in a training population of 141 genotypes were used in a multivariate genomic analysis with genome‐wide single nucleotide polymorphism data from the African bean panel. Moderate to high genomic heritability was found for GY (0.45 ± 0.10), CKT (0.50 ± 0.15), Fe (0.57 ± 0.12), and Zn (0.61 ± 0.13). There were significant favorable genetic correlations between Fe and Zn (0.91 ± 0.06), GY and Fe (0.66 ± 0.17), GY and Zn (0.44 ± 0.19), CKT and Fe (−0.57 ± 0.21), and CKT and Zn (−0.67 ± 0.20). Optimal contributions selection (OCS), based on economic index of weighted GEBV for each trait, was used to design crossing within four market groups relevant to East Africa. Progeny were predicted by OCS to increase in mean GY by 12.4%, decrease in mean CKT by 9.3%, and increase in mean Fe and Zn content by 6.9 and 4.6%, respectively, with low achieved coancestry of 0.032. Genomic selection with OCS will accelerate breeding of high‐yielding, biofortified, and rapid cooking African common bean cultivars.

## Core Ideas

- Common bean varied in grain yield, cooking time, iron, and zinc in East African field trials.
- Significant genomic heritability was present for all traits in an African bean panel.
- Favorable genetic correlations were found between high grain yield and high iron and zinc.
- Favorable genetic correlations were found between short cooking time and high iron and zinc.
- High genetic gains were predicted with genomic selection and optimal contributions selection.

**Abbreviations**
BLUE: best linear unbiased estimates
BLUP: best linear unbiased predictions
CIAT: Alliance of Bioversity International and International Centre for Tropical Agriculture
CKT: cooking time
GBLUP: genomic best linear unbiased prediction
GEBV: genomic estimated breeding values
GH: growth habit
GHP: growth habit of plot
GHT: growth habit of trial
GID: germplasm identifier number
GP: gene pool
GRM: genomic relationship matrix
GS: genomic selection
GY: grain yield
MG: market group
OCS: optimal contributions selection
PABRA: Pan Africa Bean Research Alliance
SNP: single nucleotide polymorphism
VC: variance component.

## 1 INTRODUCTION

Cooked grains of common bean (*Phaseolus vulgaris* L.) are a staple food in East Africa, where the grain protein, iron (Fe), and zinc (Zn) content are important for the health and well‐being of African people, in particular women and children (Stevens et al., 2013). Common bean production and productivity has not achieved its potential in many African countries due to production and consumption constraints, and, as a result, the health‐promoting qualities of beans are not benefiting populations that need them the most (Foyer et al., 2016).

One of the main constraints to consumption of common beans in Africa is the long time required for soaking and cooking, which also induces nutrient leaching (Wiesinger et al., 2016). Over 85% of the rural and urban poor use wood or charcoal as a source of fuel to cook beans, which is in short supply and has environmental impact due to deforestation. In many villages, firewood must be collected by women and children, and this is a dangerous and time‐consuming activity (Shellie‐Dessert & Hosfield, 1990). Nutritional well‐being and consumption should increase if bean cultivars were available that require less time and water to cook (Shellie‐Dessert & Hosfield, 1990). Bean cultivars with shorter cooking time (CKT) will result in less smoke inhalation by women burning biofuels, which will provide a major health benefit (World Health Organization, 2002). Studies show that African women prefer beans with shorter CKT (Assefa et al., 2005; Katungi et al., 2015) and are willing to pay more for short CKT and high Fe even though they are invisible traits (Rubyogo et al., 2019).

Research on “cookability” in *Phaseolus* beans began in the 1960s. Cooking time and rate of water absorption in beans were found to be unique traits (Jackson & Varriano‐Marston, 1981), with a low phenotypic correlation between the two traits and a relatively low genotype × environment interaction variance for both traits (Shellie & Hosfield, 1991). Wide genotypic variability associated with CKT existed in both Andean and Mesoamerican bean accessions (Cichy et al., 2015; Ribeiro et al., 2021; Ribeiro, Rodrigues, et al., 2014), and there was high narrow‐sense heritability of CKT in beans (*h* ^2^ = 0.74) (Jacinto‐Hernandez et al., 2003). Variation in CKT was associated with seed qualities like color, size, and seed coat thickness (Bassett et al., 2020; Kläsener et al., 2019; Reyes‐Moreno et al., 1993).

Genetic variation also existed for nutritional composition of cooked beans, including Zn, phosphorus (P), potassium (K), calcium (Ca), and Fe, in the Andean Diversity Panel (Katuuramu et al., 2018). High diversity for Fe and Zn content and grain yield (GY) was found in east and central African beans (Amongi et al., 2018; Kimani & Warsame, 2019; Mughi et al., 2021). The Manteca yellow bean was shown to be particularly rapid cooking with high Fe bioavailability, and shorter CKT was correlated with improved Fe bioavailability (Wiesinger et al., 2018).

Genomic information is an important breeding tool to improve selection accuracy and to accelerate genetic gain in beans (Delfini et al., 2021; Diaz et al., 2021; Keller et al., 2020). However, selection for desirable traits also results in erosion of genetic diversity (Esquinas‐Alcázar, 2005; Trucchi et al., 2021) and inefficient selection procedures in crop breeding may lead to premature plateauing of genetic gain (Cowling et al., 2017). The rate of erosion of genetic diversity in breeding programs may be reduced and selection efficiency improved through optimal contributions selection (OCS), which balances genetic gain against loss in genetic variation (Cowling et al., 2019, 2017; Gorjanc et al., 2018; Kinghorn, 2011; Kinghorn & Kinghorn, 2021; Woolliams et al., 2015).

Here we evaluate the potential of a diverse African common bean panel of 358 genotypes for future genetic improvement in GY, CKT, Fe, and Zn based on genomic best linear unbiased prediction (GBLUP). Selection was based on an optimized index of genomic estimated breeding values (GEBV) for each trait weighted to achieve the desired outcomes in the next generation (Brascamp, 1984; Cowling et al., 2017; Kinghorn, 2021), and OCS was used to balance genetic gain for the index under the constraint of a maximum permissible target inbreeding rate (Kinghorn & Kinghorn, 2021; Woolliams et al., 2015).

**FIGURE 1:**
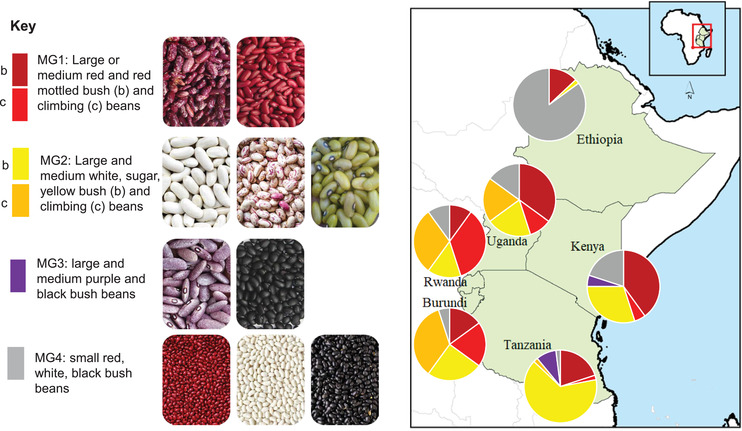
Four major market groups (MG) and their market proportions (pie charts) in six focus countries in East Africa, as proposed by breeders of common bean in the Pan Africa Bean Research Alliance. Market groups are amalgamations of market classes of common bean in Africa (Farrow & Muthoni‐Andriatsitohaina, 2020) for breeding purposes. MG1 and MG2 encompass both bush (b) and climbing (c) growth habit, as both types are grown in East Africa; MG3 and MG4 are found only as bush beans. Market proportions are based on the national common bean breeding program prioritization (C. Mukankusi, personal communication, 1 October 2020)

Genomic predictions for GY, CKT, Fe, and Zn in the African bean panel were based on a two‐stage analysis, following the approach in Sarinelli et al. (2019) and Hayes et al. (2021). In the first stage, best linear unbiased estimates (BLUE) were obtained for a training population for each trait across 33 field trials in East Africa; in the second stage, GEBV were predicted in the African bean panel from a multivariate GBLUP mixed model analysis based on BLUE from the training population and a genomic relationship matrix (GRM) from whole genome markers in the African bean panel. We then evaluated different crossing designs in OCS while allowing for genotype groupings based on bush or climbing growth habit (GH), Andean or Mesoamerican gene pool (GP), or four market groups (MGs) (Figure 1). Each MG combines several market classes of beans in Africa (Farrow & Muthoni‐Andriatsitohaina, 2020) in line with bean breeding objectives of Pan Africa Bean Research Alliance (PABRA) (Buruchara et al., 2011; Mukankusi et al., 2019). Various selection scenarios were evaluated in OCS to predict which approach would result in the greatest genetic gain in GY, CKT, Fe, and Zn.

Breeding values generated by BLUP or GBLUP, rapid cycles, index selection, and OCS are the basis of a crop breeding system implemented in a new project in East Africa (ACIAR Research, 2021) with the aim of selecting rapid‐cooking, micronutrient‐rich, and high‐yielding common bean cultivars. This paper describes a process for selecting candidates for crossing from the African bean panel to begin this project.

**Core Ideas**

- Common bean varied in grain yield, cooking time, iron, and zinc in East African field trials.
- Significant genomic heritability was present for all traits in an African bean panel.
- Favorable genetic correlations were found between high grain yield and high iron and zinc.
- Favorable genetic correlations were found between short cooking time and high iron and zinc.
- High genetic gains were predicted with genomic selection and optimal contributions selection.

## 2 MATERIALS AND METHODS

### 2.1 Field trials

Phenotypic data were obtained from 898 germplasm entries (each with a unique germplasm identifier number, GID) of bush and climbing types of common bean (Supplemental Table S1) evaluated in 33 field trials of common bean grown in different years (2015 to 2018) and different locations in East Africa. The locations were Kawanda (0°25′ N, 32°31′ E, elevation 1,190 m asl) and Kachwekano (1°15′ S, 29°57′ E, elevation 2,200 m asl) in Uganda, and Kagera (2°08′ S, 33°26′ E, elevation 1,320 m asl) in Tanzania. Germplasm entries included released cultivars and landraces common in East Africa, plus breeding lines from the Alliance of Bioversity and International Centre for Tropical Agriculture (CIAT) Kawanda, Uganda, and CIAT Colombia, and included both bush and climbing GH and diverse genetic origins in both the Andean and Mesoamerican gene pools (Bitocchi et al., 2013) (Supplemental Table S1). From the 33 field trials, GY (kg ha^−1^) was available from 32 trials, Fe and Zn (mg kg^−1^) from 29 trials, and CKT (min) from 14 trials (Supplemental Table S2). The 33 field trials were classified as either “bush” or “climbing” GH based on the GH of the majority of genotypes in the trial (GHT). In addition, GH was scored on plots (GHP) on a scale of 1 (fully bush‐type) to 4 (fully climbing‐type) (Singh, 1981). Both bush and climbing genotypes were present in all trials and there was low to moderate concurrence of genotypes across trials (Supplemental Table S3).

The field trials were designed with two or three complete blocks, subdivided into incomplete subblocks in an alpha‐lattice design, and occasionally local cultivars were replicated within blocks. Each plot consisted of three rows 3 m long and 50 cm apart with 50 cm between plots (effective plot area 4.5 m^2^). One hundred seeds were sown in each plot to achieve a target density of 90 plants after emergence. Harvested seed from each plot was cleaned, sun‐dried to approximately 13% moisture content, weighed and stored, and the weight was converted to GY based on the effective plot area.

### 2.2 Laboratory evaluation

Twenty‐five seeds were randomly picked from clean harvested seed from each plot and used to measure CKT in an automated Mattson cooker (Canadian Grain Commission) (Proctor & Watts, 1987). The 25 seeds were placed in a container of water to soak for 18 h. Twenty‐five soaked seeds were placed on a rack with sharp pins (plungers) placed on top of each seed, and the rack was submerged into hot water that was kept at 98 °C on a hotplate. Cooking time (min) was the time elapsed from the time of immersion in hot water until the 20th seed was pierced by a steel pin (Wang & Daun, 2005).

For measurement of Fe and Zn content, 50 g of dry seed per plot were cleaned in distilled water, oven‐dried at 60 °C for at least 12 h, and ground into a powder using a Sunbeam Conical Burr Mill EM0480 Grinder (Sunbeam) at the HarvestPlus lab in Rubona, Rwanda. The concentration of Fe and Zn (mg kg^−1^) was determined using XRF spectrometry as described by Paltridge et al. (2012).

### 2.3 Statistical analysis

All linear mixed models were implemented using ASReml‐R (Butler et al., 2017). Grain yield, Fe, Zn, and CKT were first analyzed in single field trials to assess repeatability of genotype performance within trials. Genomic prediction across trials followed a two‐stage analysis based on the approach of Sarinelli et al. (2019) and Hayes et al. (2021), as described below.

### 2.4 Repeatability in single trials

Repeatability of genotype performance in 33 field trials in East Africa was evaluated for each trait. The general form of the linear mixed model for GY, CKT, Fe, and Zn in each trial was

(1) Y=mean+genotype+block+block×subblock+e

where *Y* 
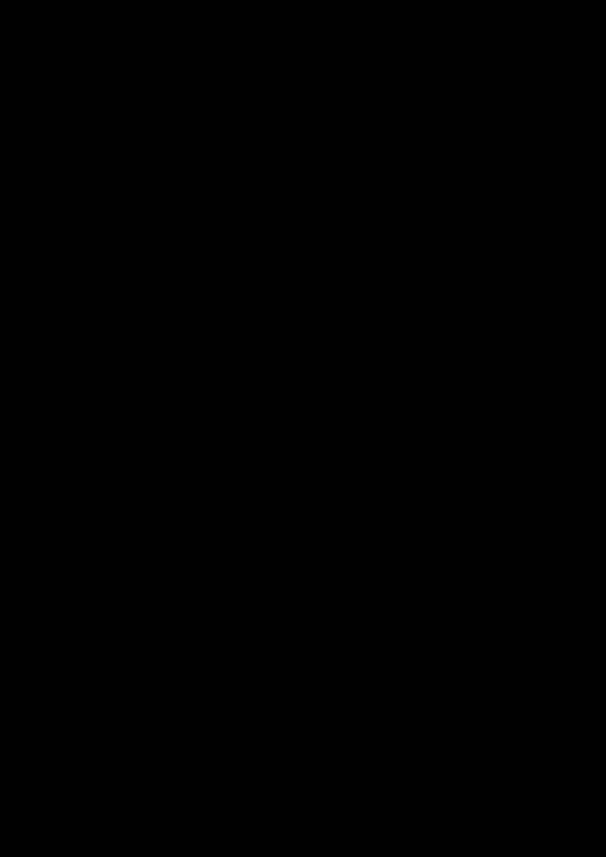
is either GY, CKT, Fe, or Zn, *block* is a complete block containing at least one replicate of genotypes (normally one block at front and one block at rear of trial), *block* × *subblock* represents the subdivision of blocks into incomplete subblocks in an alpha‐lattice design, and *e* is the random residual. In this analysis, *genotype*, *block*, and *block* × *subblock* were assumed to be random effects. Repeatability of measurement means for each trait in each trial ($R_{n}$) followed Equation 38 in Nakagawa and Schielzeth (2010) for replicated field trials:

(2) $$R_{n}=\frac{VC_{g}\;}{VC_{g}+\frac{VC_{e}}{n_{0}}}$$

where *VC*_g_ is the variance component for genotypes, *VC*_e_ is the variance component for error, and *n*_0_ is the adjusted mean number of measurements per genotype (Nakagawa & Schielzeth, 2010).

### 2.5 Stage 1 analysis: Multienvironment univariate analysis with genotype as a fixed effect

In Stage 1 analysis, BLUE were obtained for up to 898 genotypes from an analysis of GY, CKT, Fe, and Zn across 33 field trials in a univariate multienvironment BLUE mixed model analysis based on the following base formula:

(3) $$\begin{array}{c} Y\;=\;mean\;+\;genotype\;+\;trial\;+\;genotype\;\times \;trial\;+\;trial\;\times \;block\\ +\;trial\;\times \;block\;\times \;subblock\;+\;e \end{array}$$

where *Y* is either GY, CKT, Fe, or Zn; *genotype* is a fixed effect; *trial*, *genotype* × *trial*, *trial* × *block*, and *trial* × *block* × *subblock* are random effects; and *e* is a vector of random residual effects. The random effects *trial* × *block* and *trial* × *block* × *subblock* 
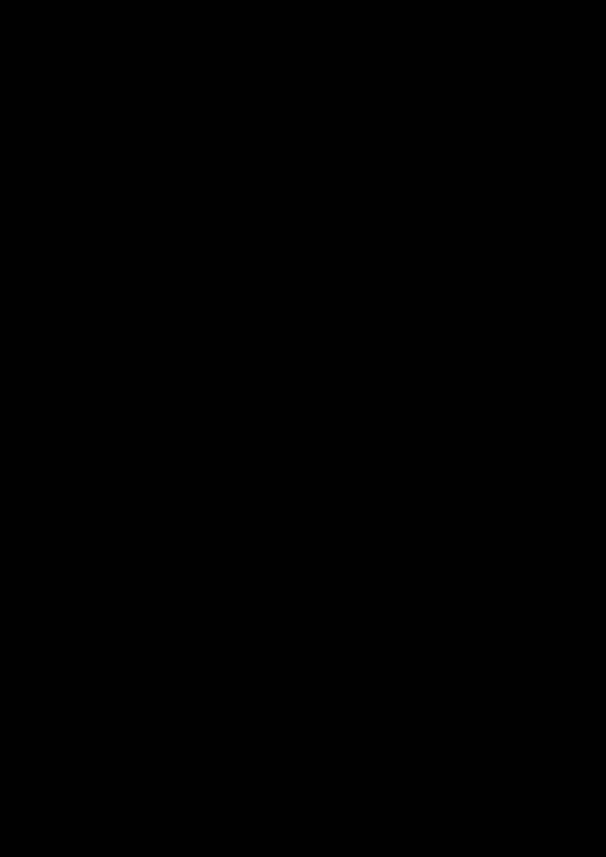
were discarded from the final model if not significant.

Three variations of the base formula of BLUE analysis were compared:

- Model 1: Base formula plus *GHT*, which was added as a fixed effect. If *GHT* was not significant, this model was discarded.
- Model 2: Base formula plus *GHP* and *GHP* × *genotype* as random effects. *GHP* was evaluated on a plot basis and *GHP* × *trial* was included because some common bean cultivars showed variable *GHP* between trials (Singh, 1981). *GHP* and *GHP* × *trial* were discarded if they were not significant in Model 2.
- Model 3: Model 2 with *GHP* and/or *GHP* × *trial* discarded if not significant in Model 2.

Significance of fixed effects was assessed by the Wald test, and the optimum model was chosen on the basis of change in Akaike information criterion (AIC), Bayesian information criterion (BIC), and log‐likelihood values.

### 2.6 Stage 2 analysis: Multivariate analysis for genomic prediction

In the second stage, BLUE of each genotype for each trait were used as values of a dependent variable in a multivariate mixed model for genomic prediction, which included a GRM for the African bean panel.

#### 2.6.1 Construction of the genomic relationship matrix

**FIGURE 2:**
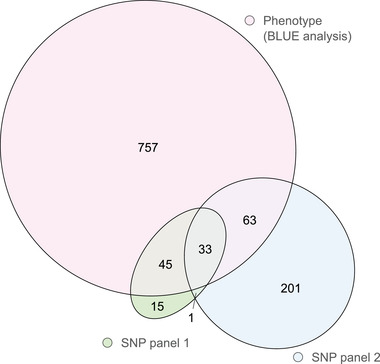
Venn diagram showing the distribution of 898 genotypes with best linear unbiased estimates (BLUE), of which 141 genotypes (the training population) were present in single nucleotide polymorphism (SNP) Panels 1 (45), 2 (63), or both 1 and 2 (33). SNP Panel 1 (94 genotypes) shared 34 genotypes in common with SNP Panel 2 (298 genotypes), and together form the African bean panel composed of 358 genotypes

A GRM was established for 358 genotypes by combining two single nucleotide polymorphism (SNP) panels (Supplemental Table S4). Panel 1 with 94 genotypes was a 770 SNP array (Raatz et al., 2019) and more than 20,000 SNPs were available in Panel 2 with 298 genotypes based on diversity array technology analyzed by the Integrated Genotyping Service and Support platform, a genotyping service facility based in Nairobi, Kenya (as used by Nkhata et al., 2020). Thirty‐four genotypes were common to both panels (Figure 2).

FImpute software (Sargolzaei et al., 2014) was used to impute missing values. The physical map position of each SNP locus in Panel 1 and Panel 2 was used to form a consensus SNP panel (defined as the African bean panel) based on 358 genotypes. The GRM of the African bean panel was calculated using the method described by VanRaden (2008).

Of the 898 genotypes evaluated in field trials, 141 were present in one or both of the SNP panels (Figure 2) and acted as the training population.

#### 2.6.2 Cluster analysis of genomic relationships in the African bean panel

Genetic relationships among the 358 genotypes in the African bean panel were assessed by cluster analysis of the GRM using R package “cluster” (Maechler et al., 2021). The genetic relationships between groups in the dendrogram were interpreted on the basis of the known Andean or Mesoamerican origin of 54 genotypes (Raatz et al., 2019).

#### 2.6.3 Estimation of GEBV by multivariate GBLUP

Best linear unbiased estimates for GY, CKT, Fe, and Zn for 141 genotypes in the training population acted as values of a dependent variable in a multivariate GBLUP analysis with the GRM to predict GEBV for 358 genotypes in the African bean panel based on the following model (in vector notation):

(4) $$\left[\begin{array}{c} y_{1}\\ \begin{array}{c} y_{2}\\ y_{3}\\ y_{4} \end{array} \end{array}\right]=\left[\begin{array}{c} 1_{n1}\mu_{1}\\ \begin{array}{c} 1_{n2}\mu_{2}\\ 1_{n3}\mu_{3}\\ 1_{n4}\mu_{4} \end{array} \end{array}\right]\;+\left[\begin{array}{c} Z_{1}\\ \;\begin{array}{c} 0\\ \;0\\ \;0 \end{array} \end{array}\begin{array}{c} 0\\ \;\begin{array}{c} Z_{2}\\ \;0\\ \;0 \end{array} \end{array}\begin{array}{c} 0\\ \;\begin{array}{c} 0\\ \;Z_{3}\\ \;0\; \end{array} \end{array}\begin{array}{c} 0\\ \;\begin{array}{c} 0\\ \;0\\ \;Z_{4} \end{array} \end{array}\right]\left[\begin{array}{c} g_{1}\\ \begin{array}{c} g_{2}\\ g_{3}\\ g_{4} \end{array} \end{array}\right]+\left[\begin{array}{c} e_{1}\\ \begin{array}{c} e_{2}\\ e_{3}\\ e_{4} \end{array} \end{array}\right]$$

where **y**_1_, **y**_2_, **y**_3_, and **y**_4_ 
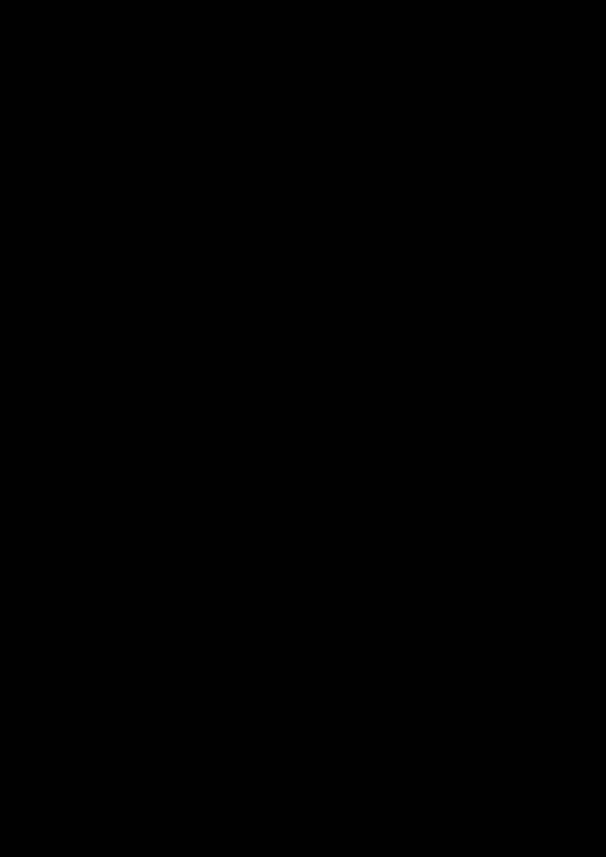
are vectors of BLUE for GY, CKT, Fe, and Zn; **1** *_n_*_i_ (*i *= 1,4) is a vector of ones for the trait *i*; **μ** *_i_* (*i *= 1,4) is overall mean for the trait *i*; **Z** *_i_* (*i* = 1,4) is a design matrix relating BLUE to genotypes for the trait *i*; **g** *_i_* is GEBV for the trait *i* with assumed distribution *N*(0, GRM **⊗ G**), where **G** is the genotypic variance‐covariance matrix for four traits; and **e** *_i_* is a vector of random residual effects for the trait *i*.

Genomic heritability ($h_{i}^{2}$) for trait *i* was estimated as follows:

(5) $$h_{i}^{2}=\frac{\sigma_{gi}^{2}}{\sigma_{gi}^{2}+\sigma_{ei}^{2}}$$

where $\sigma_{gi}^{2}$ is the additive genotypic variance for the *i*th trait and $\sigma_{ei}^{2}$ is the residual variance for the *i*th trait. This estimate of genomic heritability is subject to the limitations of GBLUP models based on whole genome inference (de los Campos et al., 2015).

Correlations of additive genetic values between traits were estimated as follows:

(6) $$\;r_{gij}=\frac{\sigma_{gij}^{2}}{\sqrt{\sigma_{gi}^{2}\sigma_{gj}^{2}}}$$

where *r_gji_* is the genetic correlation between trait *i* and *j*; $\sigma_{gij}^{2}$ is the genetic covariance between trait *i* and *j*; and $\sigma_{gi}^{2}$ and $\sigma_{gj}^{2}$ are the additive genetic variances for trait *i* and *j*, respectively.

Phenotypic correlations between traits were estimated as:

(7) $$r_{pij}=\frac{\sigma_{gij}^{2}+\;\sigma_{eij}^{2}}{\sqrt{\left(\sigma_{gi}^{2}+\;\sigma_{ei}^{2}\right)\;\left(\sigma_{gj}^{2}+\;\sigma_{ej}^{2}\right)}}$$

where $r_{pij}$ is the phenotypic correlation between trait *i* and *j*; $\sigma_{gij}^{2}$ and $\sigma_{eij}^{2}$ are the genetic and residual covariance, respectively, between trait *i* and *j*; $\sigma_{gi}^{2}$ and $\sigma_{gj}^{2}$ are the additive genetic variance for trait *i* and *j*, respectively and $\sigma_{ei}^{2}$ and $\sigma_{ej}^{2}$ are the residual variance for trait *i* and *j*, respectively.

### 2.7 Optimized selection index

We calculated a selection index for individuals in the African bean panel with economic weights on GEBV for GY, CKT, Fe, and Zn that reflected current market values for the price of beans in east Africa (US$0.85/kg) and average GY in the trials (869 kg ha^−1^), with a 15% price bonus for 10% shorter CKT and a 10% price bonus for 10% improvement in Fe and Zn content. These economic weights were optimized as implied economic weights using the tactical desired gains approach (Brascamp, 1984) in the program DESIRE (Kinghorn, 2021). DESIRE is a tool to discover possible patterns of response across traits as a function of implied economic weights. Parameters required for this analysis were accuracy and standard deviations of GEBV for traits and genetic and phenotypic correlations amongst these GEBV. The primary emphasis in DESIRE was to reduce CKT, and the secondary emphasis was to increase Fe and Zn while also improving GY, which resulted in an implied economic weight for each trait to reach the desired gains. The optimized selection index (named *GBLUPindex_i_*) was calculated for each individual *i* following the process in Cowling et al. (2017),

(8) $$\;GBLUPindex_{i}=\sum_{j=1}^{nTraits}e_{j}.GEBV_{i,j}$$

where *e_j_* is an implied economic weight for trait *j*, and *GEBV_ij_* is the GEBV for individual *i* for trait *j*.

### 2.8 OCS

Optimal contributions selection was used to generate a crossing design that balanced genetic gain for the *GBLUPindex_i_* under the constraint of a maximum permissible target inbreeding rate (Kinghorn, 2011; Kinghorn & Kinghorn, 2021; Woolliams et al., 2015). We used selection and cross allocation methods developed previously (Cowling et al., 2019; Cowling et al., 2017). Optimal contributions selection was implemented in program “MateSel” (Kinghorn & Kinghorn, 2021), which is based on an evolutionary algorithm with constraints easily invoked to ensure practical relevance and precise control of selection response and other outcomes. MateSel dictates which individuals to select and the actual crossing allocations and/or selfings to be made.

#### 2.8.1 Restricting crossing within and between groups

Genotypes in the African bean panel were classified according to GH, GP, and MG. Four MGs were chosen as a practical measure to reduce the number of market‐related groups for breeding purposes and to follow the bean breeding objectives of the PABRA (Buruchara et al., 2011; Mukankusi et al., 2019) (Figure 1). Each MG represents one or more closely related market classes of beans in Africa (Farrow & Muthoni‐Andriatsitohaina, 2020). MG1 includes large and medium‐sized red and red mottled bush and climbing beans; MG2 contains large and medium‐sized white, sugar, yellow bush, and climbing beans; MG3 consists of large and medium‐sized purple and black bush beans; and MG4 represents all small‐sized bush beans which are favored in Ethiopia (Figure 1).

The impact on predicted progeny performance of restricting crossing within groups in OCS was compared in six scenarios in OCS: (a) OCS Scenario 1 with no restriction to crossing based on groups; (b) OCS Scenario 2 with grouping on GH; (c) OCS Scenario 3 with grouping on GP; (d) OCS Scenario 4 with grouping on GH and GP; (e) OCS Scenario 5 with grouping on MGs; and (f) OCS Scenario 6 with grouping on MGs and allowing some inter‐MG crossing.

## 3 RESULTS

### 3.1 Repeatability and concurrence

Trials varied widely in mean, repeatability, and significance of genotype variance components (VCs) for GY, CKT, Fe, and Zn (Supplemental Figure S1, Supplemental Table S2). In the majority of trials, there was a significant VC for genotypes, but the VC for block was not significant in any trial and was removed from subsequent analyses. Repeatability of measurement means for GY, CKT, Fe, and Zn averaged 0.50, 0.31, 0.51, and 0.47, respectively (Supplemental Table S2). Genotype concurrence between trials varied widely, and many trials that were classified as GHT “bush” included some climbing genotypes, and vice versa (Supplemental Table S3).

### 3.2 Stage 1 analysis: Multienvironment univariate analysis with genotype as a fixed effect

Three multienvironment BLUE analysis models were evaluated. In all models and for all traits, the effects of genotype (GID, fixed effect) and trial and the interaction of trial × GID (random effects) were significant for all traits, that is, there were small changes in ranking of genotypes for all traits across trials. However, the proportion of variance accounted for by trial × GID averaged less than 20% of the variance due to trial (Supplemental Table S5).

In Model 1, GHT (fixed effect) was not significant. We verified that climbing and bush trials had similar average GY, CKT, Fe, and Zn (Supplemental Figure S1, Supplemental Table S2), and GHT was dropped from subsequent models.

In Model 2, GHP and GHP × GID were included as random effects. The VC for GHP was not significant for any trait, that is, there was no consistent difference in GY, CKT, Fe, and Zn among the four categories of GHP across all trials. However, the interaction of GHP × GID was significant for GY and Fe, that is, there were small changes in ranking of genotypes for GY and Fe across GHP. We verified that some but not all genotypes varied widely in GHP across the trials. For example, genotype MLB‐49‐89A (classified as climbing GH) varied from GHP 1 to 4 across 13 plots in 6 trials, with mean GHP 2.5. The proportion of variance accounted for by GHP × GID was always less than 10% of the variance due to trial (Supplemental Table S5).

In Model 3, the effect of removing GHP (and GHP × GID for CKT and Zn) was evaluated by assessing the change in AIC, BIC, and log‐likelihood values in the reduced models. In no case did the model improve substantially in Model 3, and hence Model 2 was selected as the final model for all four traits (Supplemental Table S5).

Based on Model 2, BLUE were estimated for 898 genotypes, of which 141 genotypes had genotypic (SNP) data (the training population). The characteristics of the training population are described in Table 1 and Supplemental Table S6.

**TABLE 1:** Summary of BLUE in the training population for grain yield per hectare (GY), cooking time (CKT), iron (Fe), and zinc (Zn) content

**Trait**	**Unit**	***n*** ^a^	**Mean**	**Min**.	**Max**.	**SD** ^b^
GY	kg ha^−1^	140	869.3	439.5	1541.3	236.3
CKT	min	117	65.3	46.1	98.5	11.0
Fe	mg kg^−1^	135	69.0	56.5	97.3	6.4
Zn	mg kg^−1^	135	35.6	30.6	43.3	2.0

*Note*: The training population consisted of 141 genotypes with both phenotypic and genotypic data, for which best linear unbiased estimates (BLUE) were estimated for *n* genotypes with phenotypic data for each trait.

^a^ *n* = number of genotypes.

^b^ SD = standard deviation.

### 3.3 Stage 2 analysis: Multivariate GBLUP analysis with genotype as a random effect

SNP Panel 1 (773 alleles) and SNP Panel 2 (22,890 alleles) were combined in a consensus panel of 21,575 markers for 358 genotypes based on the physical map position for each SNP locus to form the African bean panel. The genomic relationship matrix for the African bean panel is shown in Supplemental Table S4.

Multivariate GBLUP analysis in the African bean panel revealed moderate to high genomic heritability for all traits and a wide range of GEBV values for GY, CKT, Fe, and Zn among 358 genotypes (Table 2; Supplemental Table S7). Significant additive variance occurred for GY, CKT, Fe, and Zn. Significant positive covariance occurred between Fe and GY, Zn and GY, and Zn and Fe, and significant negative covariance between Fe and CKT and Zn and CKT (Supplemental Table S8).

**TABLE 2:** Summary of genomic estimated breeding values (GEBV) and narrow‐sense heritability (*h* ^2^) for 358 bean genotypes in the African bean panel, including grain yield per hectare (GY), cooking time (CKT), iron (Fe), and zinc (Zn) content

**Trait**	**Unit**	***n*** ^a^	**Mean**	**Min**.	Max.	**SD**	***h* ^2^ ± SE**
GY	kg ha^−1^	358	0	−222.05	460.62	119.72	0.45 ± 0.10
CKT	min	358	0	−16.06	24.63	5.17	0.50 ± 0.15
Fe	mg kg^−1^	358	0	−9.17	17.02	3.62	0.57 ± 0.12
Zn	mg kg^−1^	358	0	−3.91	5.64	1.16	0.61 ± 0.13

^a^ *n* = number of genotypes.

The genetic correlation of Fe and Zn was very high (*r* = 0.91 ± 0.06), and there were positive and significant genetic correlations between GY and Fe, and GY and Zn (Table 3). There were negative but favorable genetic correlations between CKT and Fe, and CKT and Zn, but no significant genetic correlation between GY and CKT (Table 3).

**TABLE 3:** Pairwise genetic (above diagonal) and phenotypic (below diagonal) correlations of genomic estimated breeding values (GEBV) for grain yield per hectare (GY), cooking time (CKT), iron (Fe), and zinc (Zn) content (standard error in brackets)

**Trait**	**GY**	**CKT**	**Fe**	**Zn**
GY		−0.16 (0.23)	0.66 (0.17)	0.44 (0.19)
CKT	−0.25 (0.10)		−0.57 (0.21)	−0.67 (0.20)
Fe	0.13 (0.09)	−0.23 (0.10)		0.91 (0.06)
Zn	0.15 (0.09)	−0.22 (0.10)	0.64 (0.05)	

Phenotypic correlations followed the same pattern as additive genetic correlations but were less pronounced except for the significant positive phenotypic correlation (*r* = 0.64 ± 0.05) between Fe and Zn (Table 3).

### 3.4 Genomic relationships

Cluster analysis based on SNP data for 358 genotypes revealed groups and subgroups that conformed with the known Andean or Mesoamerican origins of the African bean panel. Several genotypes in a previous study (Raatz et al., 2019) acted as reference genotypes to assist in classifying the clusters (Supplemental Figure S2, Supplemental Table S9). The reference genotypes in the upper branch belonged to the Andean gene pool and defined this cluster as “Andean” (Supplemental Figure S2). The uppermost cluster of the lower branch had several Andean reference genotypes, and this subgroup was classified as “Andean admixture.” The lower branch of the lower group had many Mesoamerican reference genotypes and hence defined this cluster as “Mesoamerican.” A branch above the Mesoamerican cluster included progenies of a cross between a Mesoamerican line (MLB‐49‐89A, GID 121058, in this branch) and an Andean genotype CAL96 (C. Mukankusi; personal communication, 9 October 2020), and this subgroup was classified as “Mesoamerican admixture” (Supplemental Figure S2). All Andean and Mesoamerican reference genotypes were aligned with respective clusters except one which was previously considered to be Mesoamerican (SCR15; GID 114370), but it was grouped with Andean reference genotypes. This may be the result of a sampling error or labeling error at some point, since the parents of SCR15 (SER118 and NCB226) are Mesoamerican.

### 3.5 Optimized selection index and OCS

Implied economic weights for each trait to reach the desired gains were 0.0014 (GY), −0.145 (CKT), 0.2416 (Fe), and 0.0058 (Zn). These implied economic weights were used as multipliers of GEBV for each trait to calculate the optimized selection index for 358 genotypes in the African bean panel (Supplemental Table S7).

Optimal contributions selection provided predicted outcomes for six crossing scenarios, where crossing was either unrestricted among the 358 candidate genotypes or restricted to within groups defined by GH, GP, or MG (Table 4, Supplemental Tables S8, S9). To evaluate the impact of restriction of mating within two GP, the Andean admixture group was merged with Andean group, and the Mesoamerican admixture group was merged with the Mesoamerican group. Two genotypes with unknown GH and seeds not available (MLAMA 127 and MEXICO222) were not considered as candidates for crossing, thereby reducing the number of candidates available for OCS to 356 (Table 4, Supplemental Table S9).

**TABLE 4:** Distribution of 356 genotypes in the African bean panel across four market groups (MGs) based on seed color and seed size, and the growth habit (GH; bush or climbing) and gene pool (GP; Andean or Mesoamerican) of genotypes within each MG

				**GH**		GP	
**MG** ^a^	**Seed size**	**Seed color**	**No. genotypes**	**Bush**	**Climbing**	**Andean** ^b^	**Mesoamerican** ^c^
MG1	Large or Medium	Red, red mottled	88	76	12	53	35
MG2	Large or Medium	White, cream, sugar, yellow	95	48	47	72	23
MG3	Large or Medium	Black, purple	80	45	35	41	39
MG4	Small	Diverse colors	64	58	6	16	48
Other	Not known	Not known	29	18	11	16	13
Total			356	245	111	198	158

^a^ “Other” includes genotypes for which seed size and color are not known.

^b^ “Andean” gene pool includes the Andean cluster group (97 genotypes) and Andean admixture cluster group (101 genotypes) (Supplemental Figure S2).

^c^ “Mesoamerican” gene pool includes the Mesoamerican cluster group (112 genotypes) and Mesoamerican admixture cluster group (46 genotypes) (Supplemental Figure S2).

A total of 120 crossings were permitted from the 356 candidates in each OCS crossing scenario. High emphasis was applied in MateSel to minimize achieved coancestry in progeny in combination with moderate genetic gains in the selection index. The number of crossings targeted within each group was proportionate to the number of candidates available in the groups (Supplemental Table S10).

The OCS Scenario 1 (no restriction on crossing based on groups) had the highest predicted progeny index but also the highest achieved coancestry. There was no advantage for CKT to allow unrestricted crossing in OCS Scenario 1 (Supplemental Table S10).

The OCS Scenario 2, which restricted crossings to within bush and climbing GH, had lower predicted genetic gain for all traits than Scenario 1. The OCS Scenario 3, which restricted crossings to within Andean and Mesoamerican GP, improved the result over Scenario 2. The weakest genetic gain was predicted for OCS Scenario 4, where crossing was restricted to within Andean bush, Mesoamerican bush, Andean climbing, and Mesoamerican climbing. The OCS Scenario 5, where crossing was restricted within MG, provided a strong predicted genetic gain in CKT, and OCS Scenario 6, which was similar to OCS Scenario 5 but permitted some crossings between MG, resulted in the highest predicted genetic response of −6.09 minutes for CKT and second highest for progeny index, GY, Fe, and Zn (Supplemental Table S10).

Eighty‐one unique genotypes were selected as the founder parents from the African bean panel from OCS Scenario 6 (Supplemental Table S11). Out of these 81 founder parents, 31 were members of the training population with both field trial and genomic data, and the remaining 50 were selected on the basis of genomic data only. From the 120 crossings designed by MateSel in OCS Scenario 6, 77 were within MG, 33 were between MG, and 10 involved “other” genotypes for which seed size and color were not known (Table 5, Supplemental Table S10).

**TABLE 5:** Number of crosses within and between four market groups (MGs), gene pools (Andean or Mesoamerican), and growth habits (bush or climbing), and sex of the parent varieties as allocated by MateSel in optimum contributions selection Scenario 6 (Supplemental Table S10)

		**Female parent**									
		**Market groups**					**Gene pool**		**Growth habit**		**Total**
		**MG1**	**MG2**	**MG3**	**MG4**	**Other**	**Andean**	**Mesoamerican**	**Bush**	**Climbing**	
Male parent	MG1	21	2	2	0	0					
	MG2	5	25	6	7	1					
	MG3	2	3	18	2	2					
	MG4	2	2	0	13	1					
	Other	0	0	1	0	5					120
	Andean						33	59			
	Mesoamerican						23	5			120
	Bush								37	22	
	Climbing								31	30	120

*Note*: The MG, gene pool, and growth habit of cross parents are shown in Supplemental Table S11. Matesel allocates parents as either male or female.

In OCS Scenario 6, there was no restriction on crossing design based on GP, but 82 of the 120 proposed crosses were Andean × Mesoamerican gene pools, 33 were Andean × Andean, and five were Mesoamerican × Mesoamerican (Table 5). Likewise, there was no restriction on crossing design based on GH, but 53 of the proposed crosses were bush × climbing types, 37 were bush × bush types, and 30 were climbing × climbing types (Table 5). Optimal contributions selection favored inter‐GP and inter‐GH matings, which maximized predicted genetic gain in progeny index for a low level of achieved coancestry (Supplemental Table S11).

Strong genetic gain in mean progeny performance was predicted in OCS Scenario 6, with 12.4% increase in GY, 6.9% increase in Fe, 4.6% increase in Zn, and 9.3% decrease in CKT (Table 6) with a mean parental coancestry of 0.032 (Supplemental Table S11). The strongest genetic gain in CKT, Fe, and Zn was predicted in the unknown seed color group “other”.

**TABLE 6:** Average predicted mean genetic gain in progeny of crosses designed by optimal contributions selection (OCS) Scenario 6 (Supplemental Table S10) within four market groups (MGs) and an unclassified group (Other), between groups (Intergroup), and across the whole population of 120 crosses in the African bean panel

		**Mean predicted genetic gain in progeny of crosses designed by OCS Scenario 6**								
		**Within and between MGs**						**Whole population**		
**Traits**	**Units**	**MG1**	**MG2**	**MG3**	**MG4**	**Other**	**Inter group**	**Average of 120 cross progeny**	**Population mean BLUE (** **Table** **1** )	**Percent gain or loss**
GY	kg ha^−1^	32.41	219.46	62.91	72.02	198.81	154.77	107.61	869.33	12.38
CKT	min	−4.85	−6.10	−6.34	−5.70	−10.94	−7.80	−6.09	65.33	−9.32
Fe	mg kg^−1^	2.88	7.66	3.36	2.86	9.60	6.91	4.73	68.99	6.86
Zn	mg kg^−1^	1.12	2.06	1.46	1.12	3.47	2.24	1.62	35.58	4.55

*Note*: The unclassified group (Other) had unknown seed attributes. Percent gain or loss is expressed relative to the population mean BLUE, from Table 1). BLUE, best linear unbiased estimates; CKT, cooking time; Fe, iron; GY, grain yield; Zn, zinc.

## 4 DISCUSSION

In this study, we used a two‐stage analysis to predict GEBV for 358 common bean genotypes in an African bean panel for GY, CKT, Fe, and Zn. Genomic estimated breeding values were derived from a multivariate mixed model for genomic prediction involving a genomic relationship matrix for the African bean panel, and BLUE for GY, CKT, Fe, and Zn for 141 genotypes in a training population that acted as values of a dependent variable. For the first time in common bean breeding, we implemented OCS to specify crosses which optimized a function of genetic diversity and genetic gain in an optimized selection index (Kinghorn & Kinghorn, 2021; Woolliams et al., 2015). Progeny of crosses designed by OCS in four MGs relevant to East African markets were predicted to increase in mean GY by 12.4%, decrease in mean CKT by 9.3% and increase in mean Fe and Zn content by 6.9 and 4.6%, respectively, with a low achieved coancestry of 0.032 (Table 6).

Stochastic modelling with OCS in self‐pollinating crop breeding programs (Cowling et al., 2019, 2017) demonstrated rapid and sustained future genetic gain in an economic index for multiple traits with similar heritability and genetic correlations as found in this bean study. Based on the previous modelling, OCS in this study should result in sustainable genetic gain in GY, CKT, and Fe and Zn in breeding programs in East Africa for many decades to come.

The African bean panel included both bush and climbing types which varied in maturity date, but plot maturity date was not collected in the field trials. Climbing beans generally mature later and have higher yield potential than bush types (Checa & Blair, 2012; Katungi et al., 2018) and were considered to be more suitable for Fe and Zn biofortification (Blair, 2013). In our data, GHT was not a significant fixed effect in BLUE analysis of GY, CKT, Fe, or Zn; that is, mean GY, CKT, Fe, and Zn were the same for all trials whether they were nominated as bush or climbing trials. Therefore, GHT was removed from the model. However, there was a small but significant interaction of GHP × GID (the interaction between plot rating for GH and genotype) for GY and Fe, and GHP and GHP × GID were retained in the BLUE model for those traits (Supplemental Table S5). Growth habit of plot varied from plot to plot and trial to trial for a particular genotype. A genotype which expressed a climbing GH in one plot may have had higher Fe and GY relative to other plots where it expressed a bush GH. Growth habit of plot varied widely within and between trials in several genotypes such as MLB‐49‐89A, KK8, and CNF5520 in our dataset, and this variation in GHP may be responsible for the significant GHP × genotype interaction for Fe and GY.

The African bean panel included candidates from the Andean and Mesoamerican gene pools. Genetic differentiation between the Andean and Mesoamerican bean accessions has been well documented (Delfini et al., 2021; Lobaton et al., 2018; Raatz et al., 2019). Many Andean beans have higher Fe concentration than Mesoamerican beans (Blair, 2013; Blair et al., 2010).

The moderate‐to‐high genomic heritability values for GY (0.45), CKT (0.50), Fe (0.57), and Zn (0.61) in our study confirms the potential for future genetic improvement in these traits in the African bean panel. High heritability values were reported previously for CKT (Elia et al., 1997; Jacinto‐Hernandez et al., 2003) and Fe (Jost et al., 2013; Mukamuhirwa et al., 2015; Zemolin et al., 2016).

We also found several favorable genotypic and phenotypic correlations in the African bean panel (Table 3). Iron and Zn concentrations in seed were highly positively correlated as reported previously (Cichy et al., 2009; Hummel et al., 2020; Mukamuhirwa et al., 2015). Low‐to‐moderate positive phenotypic correlations between Fe and Zn, and between Fe or Zn and GY, were reported by Gelin et al. (2007). Glowacka et al. (2015) also reported a positive correlation between Zn and GY, but a negative correlation between Fe and GY. Other studies report weak negative correlation or no correlation between Fe or Zn with GY (Dias et al., 2021; Jost et al., 2013; Ribeiro, Jost et al, 2014). Ribeiro, Jost et al. (2014) identified some genotypes with superior GY and Fe despite a weak negative correlation between these traits. Phenotypic and genetic correlations of GY with Fe and Zn thus appear to be specific to experimental populations and environments (Dias et al., 2021; Ribeiro, Jost et al., 2014), and phenotypic correlations are not fully translated into genotypic correlations (Searle, 1961) as we observed in our study (Table 3).

The favorable genetic correlations between CKT and Fe or Zn in uncooked seed in our study contrasts with previous studies where phenotypic correlations were close to zero (Wiesinger et al., 2018; Mughi et al., 2021). It has been reported that rapid‐cooking genotypes retain more Fe and Zn and the Fe is more bioavailable than in slow‐cooking genotypes (Wiesinger et al., 2018, 2016). The negative genetic correlations between CKT and Fe or Zn in the African bean panel will help to improve genetic progress for breeding rapid‐cooking biofortified bean cultivars, which should retain more Fe/Zn after cooking. We expect genetic correlations between CKT, Fe, Zn, and GY in the African bean panel will change over cycles of breeding as allele frequencies change (Falconer & Mackay, 1996), and therefore genetic correlations must be re‐assessed in every cycle.

The effect of environment (trial) as a random effect was highly significant on all four traits in this study, although the VCs for trial × GID (equivalent to genotype × environment or G × E interaction) were relatively minor compared to the VCs for trial (Supplemental Table S4). Low G × E interaction for seed Fe was reported by Beebe et al. (2000) and Cichy et al. (2019). Moderate G × E interaction was reported for CKT in beans (Proctor & Watts, 1987) although the effect of E was minor compared to the effect of G (Jacinto‐Hernandez et al., 2003; Katuuramu et al., 2020; Shellie & Hosfield, 1991). Cichy et al. (2019) reported that G × E for CKT was low; thus, only a small number of field trials are needed to select for rapid‐cooking genotypes. In contrast, seed storage environment, time, conditions, and soaking time affected CKT measurements greatly (Arruda et al., 2012; Jackson & Varriano‐Marston, 1981; Stanley, 1992). Therefore, selection experiments for CKT must be based on standardized storage conditions and soaking time.

Comparison of different selection scenarios under OCS showed that maximum predicted genetic gain in progeny index was achieved when no restrictions were applied to the crossing design (Supplemental Table S10). However, the alliance of bean breeders and researchers in Africa (PABRA) considers that it is important to breed for MGs based on seed color and size (Buruchara et al., 2011; Mukankusi et al., 2019). Optimal contributions selection can guide breeders toward a favorable outcome in terms of breeding goals and rates of inbreeding and helps to answer questions, such as whether crossing should be done between different MG (mixing seed color and size), different GH (mixing bush and climbing types), or different GP (mixing Andean and Mesoamerican types). White et al. (1992) suggested exploitation of genetic variance in climbing types to improve potential yield in bush types. MateSel can be used as a tool to discover the best outcome in the breeding program based on various technical and logistical constraints (Kinghorn & Kinghorn, 2021). One technical constraint may be potentially poor field performance in progeny of Andean bush × Mesoamerican bush types due to a low frequency of shared domestication genes in the two GP (Schmutz et al., 2014). If this occurs, then several cycles of recurrent selection under OCS should re‐establish a high frequency of favorable domestication alleles.

The best result from OCS with MateSel, aligned with the breeding goals of PABRA, was obtained when genotypes were grouped based on MGs and some intergroup crossings were permitted in OCS Scenario 6 (Supplemental Table S10). Scenario 6 resulted in several crossings between bush and climbing types (Table 5). Segregation will occur in progeny of Scenario 6 for GH, seed color, and seed size. This project is linked into regional breeding programs in six partner countries in East Africa where selection for these traits will occur during selfing generations, and segregation for these traits in early generations may be an advantage because each breeding program has slightly different goals and target agro‐ecological environments and target markets. Some breeders seek to release both bush and climbing cultivars with locally preferred seed colors and size, whereas others are focused mostly on small‐seeded bush beans with a range of seed colors (Figure 1).

Scenario 6 also resulted in a high proportion (68%) of crosses between the two major gene pools of common bean, Andean and Mesoamerican. Only 4 and 28% of designed crosses were within the Mesoamerican and Andean pool, respectively. The drivers of OCS are to promote strong genetic gain in index while controlling for a low rate of population inbreeding (Woolliams et al., 2015), and in this study this resulted in a high proportion of inter‐GP crosses. There is strong evidence from this and other studies (Raatz et al., 2019) that genetic distance between the two pools is high, but disagreement on the degree of genetic diversity within pools (Bitocchi et al., 2013; Bitocchi et al., 2017; Kwak & Gepts, 2009; Talukder et al., 2010). Narrowing of genetic diversity within the Mesoamerican and Andean gene pools has been documented (Bitocchi et al., 2013), and previous studies also support the use of interpool crossing to increase the potential for future genetic improvement in CKT, Fe, and Zn (Beebe, 2020; Blair, 2013; Blair et al., 2010).

Our study demonstrates the high potential for rapid genetic improvement of the African bean panel for GY, CKT, Fe, and Zn through short cycles of genomic selection (Supplemental Figure S3). We propose to begin annual cycles of recurrent genomic selection for GY, CKT, Fe, and Zn following the principles of bulk S_2_ (S_0,2_) family selection (Walsh & Lynch, 2018), which was used to model genetic gain in GY and heat stress tolerance in wheat (*Triticum aestivum* L.) based on BLUP and OCS (Cowling et al., 2019). We use the acronym BRIO (ACIAR Research, 2021) to summarize this breeding method, which is based on accurate breeding values (B), rapid cycles (R), index selection (I), and optimized mating designs based on OCS (O). Scenario 6 (Table 6) is our preferred option to rapidly improve GY, CKT, Fe, and Zn by genomic selection over five annual cycles of bulk S_2_ family selection in four MGs of common bean (Figure 1) relevant to East Africa.

An intermediate goal of biofortification with Fe is considered to be 22 mg kg^−1^ over the local check (Beebe, 2020). In this paper, an increase in Cycle 1 progeny mean Fe of 4.7 mg kg^−1^ is predicted in OCS Scenario 6 (Table 6). If this rate of genetic gain continues over the next five annual cycles of recurrent selection, the population mean Fe will increase by more than 22 mg kg^−1^, and mean CKT will be 30‐40% faster than in the African bean panel.

## AUTHOR CONTRIBUTIONS

Renu Saradadevi: Data curation; Formal analysis; Investigation; Methodology; Project administration; Visualization; Writing‐original draft; Writing‐review & editing. Clare Mukankusi: Conceptualization; Data curation; Funding acquisition; Investigation; Methodology; Project administration; Resources; Supervision; Validation; Visualization; Writing‐review & editing. Li: Data curation; Formal analysis; Investigation; Methodology; Software; Writing‐review & editing. Winnyfred Amongi: Data curation; Investigation; Methodology; Project administration; Resources; Supervision; Writing‐review & editing. Julius Peter Mbiu: Data curation; Investigation; Methodology; Project administration; Resources; Writing‐review & editing. Bodo Raatz: Data curation; Formal analysis; Methodology; Software; Validation; Visualization; Writing‐review & editing. Daniel Ariza: Data curation; Formal analysis; Methodology; Software; Validation; Visualization; Writing‐review & editing. Steve Beebe: Funding acquisition; Methodology; Resources; Supervision; Writing‐review & editing. Rajeev K. Varshney: Conceptualization; Funding acquisition; Methodology; Validation; Writing‐review & editing. Eric Huttner: Conceptualization; Funding acquisition; Project administration; Resources; Writing‐review & editing. Brian Kinghorn: Methodology; Software; Visualization; Writing‐review & editing. Robert Banks: Methodology; Resources; Software; Writing‐review & editing. Jean Claude Rubyogo: Conceptualization; Funding acquisition; Methodology; Project administration; Supervision; Writing‐review & editing. Kadambot H. M. Siddique: Conceptualization; Funding acquisition; Project administration; Resources; Supervision; Validation; Writing‐review & editing. Wallace Andrew Cowling: Conceptualization; Data curation; Formal analysis; Funding acquisition; Investigation; Methodology; Project administration; Resources; Supervision; Visualization; Writing‐original draft; Writing‐review & editing.

## CONFLICT OF INTEREST

The authors declare no conflict of interest.

## Supporting information


**Supplemental Table S1**. A total of 898 genotypes were evaluated in one or more field trials of common bean in East Africa from 2015 to 2018 (trials summarised in Supplemental Table S2). The genotypes are listed here based on Genotype Identifier (GID) and Designation as recorded in the Breeding Management System (BMS) database for common bean (https://bmspro.io), number of field trials in which genotypes were evaluated, number of replications across trials, genomic information available in the African bean panel (see Supplemental Table S4), average growth habit rating on plot (GHP) across all replicates and trials on a scale of 1 (fully bush) to 4 (fully climbing), and seed size classified as small (less than 25 g/100 seeds), medium (between 25 and 40 g/100 seeds), and large (more than 40 g/100 seeds) in terms of average 100 seed weight as listed in BMS.
**Supplemental Table S2**. Mean, standard deviation (SD), growth habit of trial (GHT), number of blocks (b), average number of replicates of genotypes in trial (n_0_), variance components (VC) for genotype (g), error (e), blocks (b) and subblocks within blocks (sb) and repeatability for grain yield in kg ha^−1^ (GY), cooking time in min (CKT), iron content in mg kg^−1^ (Fe) and zinc content in mg kg^−1^ (Zn) of 898 genotypes grown in 33 field trials in Uganda (Ug) and Tanzania (Tz). NA = data not available. Repeatability of measurement means (*R_n_*) for each trait was calculated as *R*_n_ = VC_g_/(VC_g_ + [VC_e_/n_0_]) (Nakagawa & Schielzeth, 2010). GHT was classified as bush (B) or climbing (C) based on the GH of the majority of genotypes in the trial. Significance of VC was based on a one‐tailed Z‐test and are represented by * (α = 0.05), ** (α = 0.01), *** (α = 0.001) and NS (not significant).
**Supplemental Table S3**. Concurrence of genotypes among 33 field trials of common bean in East Africa. Values on the diagonal represents the number of genotypes in each trial. Each trial is classified as bush (B) or climbing (C) trial based on the growth habit of majority of genotypes in the trial (GHT).
**Supplemental Table S4**. Realized genomic relationship matrix from a consensus panel of 21,575 SNP markers for 358 genotypes based on the physical map position for each SNP locus. Genotypes are identified by name and germplasm identifier number (GID). The relationship matrix was calculated from the SNP information from two SNP panels combined by imputation to form the African bean panel.
**Supplemental Table S5**. Summary of multi‐trial BLUE models for grain yield (GY), cooking time (CKT), seed iron content (Fe) and seed zinc content (Zn) showing Wald statistic of fixed effects and variance components of random effects. Fixed effects were genotypes (identified by GID) and growth habit of trial (GHT) and random effects were the individual trials (Trial), growth habit of individual genotypes based on plot observations (GHP) and their interactions. Significance of fixed effects were tested by P‐value from the Wald test, and random effects were tested by significance of the one‐tailed Z‐test, and are represented by *** (α = 0.001), ** (α = 0.01), * (α = 0.05) and NS (not significant). Akaike information criterion (AIC) and Bayesian information criterion (BIC) and Loglikelihood values were used for comparing models 2 and 3.
**Supplemental Table S6**. Best linear unbiased estimators (BLUE) ± standard errors for grain yield (GY), cooking time (CKT), iron content in seed (Fe), and zinc content in seed (Zn) of the training population (141 genotypes of common bean with both genotypic and phenotypic data). Genotypes are identified by their genotype name and germplasm identifier number (GID), and GHP is the growth habit recorded on plots of each genotype averaged across field trials on a scale of 1 (fully bush) to 4 (fully climbing). BLUE were derived from the multi‐trial analysis of 898 genotypes across 33 trials in East Africa, with genotype as a fixed effect. NA = not available.
**Supplemental Table S7**. Genomic estimated breeding values (GEBV) for grain yield (GY), cooking time (CKT), seed iron content (Fe) and seed zinc content (Zn) of 358 genotypes in the African bean panel. GEBVs were calculated in a multivariate genomic best linear unbiased prediction analysis, where BLUE for 141 genotypes in the training population acted as values of a dependent variable to predict GEBV for GY, CKT, Fe and Zn. Significance of GEBV at α = 0.05, 0.01 and 0.001 are given as *, **, *** respectively. Genotypes are identified by their name and germplasm identifier number (GID). The optimised selection index was calculated as the sum across the four traits of GEBV times the implied economic weight.
**Supplemental Table S8**. Variance and covariance components and their standard errors (SE) from multivariate genomic best linear unbiased prediction analysis for grain yield (GY), cooking time (CKT), iron (Fe) and zinc (Zn) content of seeds. Significance was evaluated by one‐tailed (variance components) or two‐tailed (covariance components) Z‐test at α = 0.05 (*), 0.01 (**) and 0.001 (***).
**Supplemental Table S9**. Market group (MG), growth habit (GH) and gene pool (GP) of 358 genotypes in the African bean panel and training population. Genotypes are identified by their name and germplasm identifier number (GID). MG are defined in Figure 1 and Table 4 and genotypes with unknown MG are labelled “Other”. GP are defined in Supplemental Figure 2 and include Andean, Andean admixture, Mesoamerican, and Mesoamerican admixture. The GP of reference genotypes was published previously (Raatz et al. 2019). The occurrence of genotypes in the training population is also noted.
**Supplemental Table S10**. Summary of six scenarios for crossing based on optimal contributions selection (OCS) in the African bean panel when restrictions are placed on crossing within groups defined by growth habit (GH), gene pool (GP) or market group (MG), or there are no restrictions based on groups (Nil). In each scenario, the total number of crossings was 120, and all settings were the same for every scenario except for restrictions applied to crossing within groups and number of crossings allowed in each group. Predicted mean progeny response from OCS above or below the mean of candidate parents are shown in kg ha^−1^ for grain yield (GY), min for cooking time (CKT) and mg kg^−1^ for iron (Fe) and zinc (Zn) content of seed.
**Supplemental Table S11**. Predicted progeny index and mean progeny GEBV for grain yield (GY in kg ha^−1^), cooking time (CKT in min), iron (Fe in mg kg^−1^) and zinc (Zn in mg kg^−1^) from 120 crosses of common bean based on optimal contributions selection scenario 6 where crossing is partially restricted to within market groups (Supplemental Table S10). GEBV are relative to the population mean (869 kg ha^−1^ for GY, 65.3 min for CKT, 69 mg kg^−1^ for Fe and 35.6 mg kg^−1^ for Zn). Female germplasm identifier (GID) and male GID are used to identify the genotypes used as female and male parents, respectively, followed by their market groups, growth habits (bush or climbing) and gene pool (as shown in Supplemental Table S9). It is not necessary to strictly adhere to crossing direction in common bean, since the flowers are hermaphroditic.
**Supplemental Fig. S1**. Box plots showing mean and range of grain yield (GY, kg ha^−1^), cooking time (CKT, min), iron content in seed (Fe, mg kg^−1^) and zinc content in seed (Zn, mg kg^−1^) in 33 field trials of common bean conducted in East Africa over 4 years (see Supplemental Tables S1 and S2 for summary of field trial sites).
**Supplemental Fig. S2**. Dendrogram of genotype relationships formed by cluster analysis of SNP data from two SNP panels merged by imputation to form the African bean panel. Accessions were classified as Andean (upper blue area), Andean admixture (second green cluster from top), Meso admixture (third yellow cluster from top) and Mesoamerican genepool (lower red area) based on presence of genotypes with known gene pools in the cluster (Raatz et al., 2019). GIDs of bush genotypes are in green color and climbing in blue color.
**Supplemental Fig. S3**. Diagram of proposed breeding program with annual cycles of genomic selection, and optimum contributions selection (OCS) based on a selection index optimised for desired genetic gain in grain yield (GY), cooking time (CKT), and iron (Fe) and zinc (Zn) content of seeds. Cycle 1 begins when homozygous founder lines in the crossing list (Supplemental Table S11) and their F_1_ progeny are randomly intercrossed during two rounds of crossing. F_2_ self seeds are harvested from F_1_ parent plants and S_0_ seeds from each F_1_ × F_1_ cross. F_2_‐derived F_3_ (F_2,3_), F_2,4_, S_0,1_ and S_0,2_ bulks are produced in the contra‐season. These are phenotyped in Cycle 1 field trials for GY and in the laboratory for CKT, Fe and Zn. Breeding values are estimated for traits in genotyped F_2_ and S_0_ plants based on pedigree and/or genomic relationship information and used to develop an economic selection index. Cycle 2 begins with crossing of self progeny of selected S_0_ and F_2_ genotypes from Cycle 1 according to a mating design from OCS. Cycle 3 begins with mating of self progeny of S_0_ and S_2_ selections from Cycle 2. Near‐homozygous S_4_‐derived lines with high economic index are available for promotion to pre‐commercial testing from Cycle 2 onwards.

## Data Availability

The authors will make available the original field trial and SNP data to interested parties upon request.
